# Age Bias in Selection Decisions: The Role of Facial Appearance and Fitness Impressions

**DOI:** 10.3389/fpsyg.2017.02065

**Published:** 2017-12-08

**Authors:** Michèle C. Kaufmann, Franciska Krings, Leslie A. Zebrowitz, Sabine Sczesny

**Affiliations:** ^1^Department of Organizational Behavior, University of Lausanne, Lausanne, Switzerland; ^2^Department of Psychology, Brandeis University, Waltham, MA, United States; ^3^Department of Psychology, University of Bern, Bern, Switzerland

**Keywords:** age discrimination, facial age appearance, hireability, fitness impressions, personnel selection

## Abstract

This research examined the impact of facial age appearance on hiring, and impressions of fitness as the underlying mechanism. In two experimental hiring simulations, one with lay persons and one with Human Resource professionals, participants evaluated a chronologically older or younger candidate (as indicated by date of birth and age label) with either younger or older facial age appearance (as indicated by a photograph). In both studies, older-looking candidates received lower hireability ratings, due to less favorable fitness impressions. In addition, Study 1 showed that this age bias was reduced when the candidates provided counter-stereotypic information about their fitness. Study 2 showed that facial age-based discrimination is less prevalent in jobs with less costumer contact (e.g., back office).

## Introduction

Population aging is a major topic around the world (e.g., United Nations, 2013; The United States Census Bureau, 2014; European Union, 2015). It profoundly affects, among other things, the composition of the labor force, which is becoming more age-diverse with an increasing number of older workers. Within organizations, this age composition can increase intergenerational collaboration and lead to change and innovations, but it also can lead to conflicts by heightened generational tension (Joshi et al., 2011; North and Fiske, 2015).

In order to benefit from the age diversity and attract the best talent, organizations need to make sure that their recruitment and hiring processes are free of age bias against older workers. However, analyses of legal claims (von Schrader and Nazarov, 2015), workforce surveys (e.g., Kelly Services, 2006), and experimental as well as field studies (e.g., Gordon and Arvey, 2004; Bal et al., 2011) suggest that organizations have not yet reached this goal: discrimination against older workers is prevalent, particularly at hiring. In fact, organizations are reluctant to invest in older workers (North and Fiske, 2016) and show bias in hiring older workers (Abrams et al., 2016), especially older female workers (Neumark et al., 2015). Often, the age bias goes back to negative stereotypes about older workers that are activated when recruiters learn about the age of the job candidate (Perry et al., 1996; Shore and Goldberg, 2005). That is, older workers are viewed as being less competent than younger workers, and are thus less likely to be hired (Krings et al., 2011). This negative age stereotype persists despite evidence for the maintenance of job performance with age (Gordon and Arvey, 2004; Bal et al., 2011).

Associations between candidates’ chronological age, age stereotypes and recruiters’ hiring decisions are well-known and documented in the literature. That is, the activation of age stereotypes, based on information about a candidate’s chronological age, plays a central role in explaining age discrimination at hiring (e.g., Krings et al., 2011). However, recruiters’ impressions of job candidates are not only affected by beliefs that are activated by demographic information (i.e., the job candidate’s age) but also by trait impressions that are triggered by facial age appearance. When people perceive another person’s face, they readily and within milliseconds make inferences concerning this person’s traits and competencies, and these inferences in turn influence their judgments of and behaviors toward the person (e.g., Zebrowitz, 1996; Todorov et al., 2005; Leopold and Rhodes, 2010). There is initial evidence that an older facial appearance too has an impact on how people behave toward a target. That is, people are less likely to hire older-looking than younger-looking job candidates. More specifically, older-looking candidates were less likely to be hired than younger-looking ones, presumably because older age appearance triggered impressions of lower health and fitness (Kaufmann et al., 2016). Thus, an older facial appearance can lead to discrimination at hiring. However, several crucial elements are not well-understood yet, notably the joint effect of chronological age and facial age appearance, its underlying mechanisms and boundary conditions, and need further investigation. This is the goal of the present research.

## The Impact of Facial Age Appearance at Hiring

During the recruitment process, decision makers typically have both types of age information about a candidate at their disposal, the candidate’s chronological age and his or her facial age appearance: in most European countries, chronological age as well as facial age appearance are readily available on the résumé because candidates systematically include their date of birth as well as a photograph. This practice is very common and even obligatory in some countries (see, for example, recommendations by manpower at https://www.manpower.ch/en/candidates/advice/the-application/the-striking-cv/the-cv-content/; or by the European Union at http://www.yourfirsteuresjob.eu/en/home). If date of birth and/or a photograph are not included on the résumé, as it is common practice for example in the United States, recruiters can easily discover this information from sources like professional networks (e.g., LinkedIn or Xing) or video applications.

Accordingly, chronological age as well as facial age appearance of candidates are likely to influence decision makers’ judgments and hence need to be considered when studying age discrimination at hiring. To our knowledge, only one study has investigated the impact of these two types of information, but it examined their effects separately (Kaufmann et al., 2016). More specifically, in this experimental study, both types of age information were manipulated separately, i.e., decision makers either saw the candidate’s date of birth or photograph. Results showed that older-looking candidates had lesser chances of being selected for an interview than younger-looking candidates.

However, as pointed out above, in most personnel selection procedures, decision makers possess both types of age information. Thus, in spite of the first evidence that candidates’ facial age appearance affects selection decisions (Kaufmann et al., 2016), the key question regarding the joint or interactive effects of chronological age and facial age appearance and their relative strengths remains open. How do decision makers evaluate candidates when they see both candidates’ age as well as their facial age appearance? Will a candidate who is known to be older be discriminated against if he or she looks younger? Or will a candidate known to be younger be a target of discrimination if he or she looks older?

## Mechanisms Underlying Effects of Facial Age Appearance

Impressions based on faces have a profound impact on how people perceive and judge others. More specifically, people quickly derive impressions and infer personal attributes from sensory cues in the face (such as facial symmetry, size and placement of the eyes; Zebrowitz, 1996; Bodenhausen and Macrae, 2006; Freeman and Ambady, 2011). These impressions, in turn, may guide their behaviors (e.g., voting decisions, Olivola and Todorov, 2010; hiring decisions, Sczesny and Kühnen, 2004; avoidance of sick individuals or approaching competent people; Zebrowitz, 2011).

In general, an older physical appearance signals lack of fitness (Zebrowitz et al., 2003; Zebrowitz and Montepare, 2008). Correspondingly, impressions of overall fitness have been found to mediate the effect of older facial age appearance on hiring (Kaufmann et al., 2016). However, fitness impressions can be broken down into aspects of physical condition (e.g., healthy) and aspects of cognitive fitness (e.g., intelligent), and older-looking people are typically perceived as both less physically and less cognitively fit (e.g., Rosen and Jerdee, 1976; Montepare and Zebrowitz, 2002; Zebrowitz et al., 2003; van Dalen et al., 2010).

In work settings, cognitive fitness indicates not only worker’s ability to handle a complicated task, but also how quickly new skills can be acquired. On the other hand, physical fitness is not only important for physically demanding jobs, but also indicates the activity and endurance of an employee at work. Thus, both physical and cognitive fitness signal workers’ capability and potential productivity. Therefore, both are expected to play a role in age discrimination in hiring decisions (Landy et al., 1995). In line with this consideration, we expected decision makers to ascribe lower (physical and cognitive) fitness to older-looking candidates than to younger-looking ones.

## Overview of the Present Research and Hypotheses

The aim of the present research was to analyze the joint impact of chronological age and facial age appearance on hiring and to determine fitness impressions as underlying mechanisms. In two experimental hiring simulations, we examined whether impressions of fitness mediated effects of facial age appearance on hireability ratings. Participants assumed the role of the personnel manager and evaluated the résumé of a fictitious job candidate. To examine the joint impact of the candidate’s facial age appearance and chronological age, we manipulated the two variables in a fully crossed design: depending on the experimental condition, the candidate was either 50 years old and looking his or her age, 50 years old and looking younger, 30 years old and looking his or her age, or 30 years old and looking older.

Current models of person construal (e.g., Freeman and Ambady, 2011) provide the theoretical framework for explaining the influence of both types of age information. According to this model, person perception is the result of the joint influence of category information (e.g., demographics which may activate stereotypical beliefs; top–down processes) and information derived from sensory cues (e.g., facial features; bottom–up process). Because facial appearance is more vivid and salient information than demographic information (Zebrowitz, 1996; Leopold and Rhodes, 2010), facial age appearance may be more influential than chronological age. Accordingly, we expected a candidates’ older facial age appearance, independent of a candidates’ chronological age, to trigger less favorable ratings at hiring.

Specifically, we predicted that older-looking candidates would be perceived as less fit (Hypothesis 1a) and receive less favorable hireability ratings (Hypothesis 1b) than younger-looking ones, independent of their chronological age. Moreover, we predicted that the lower hireability ratings for older-looking versus younger-looking candidates would be mediated by impressions of lower fitness (Hypothesis 2).

In Study 1 we examined whether the impact of facial age appearance on hiring can be reduced by targeting the presumed underlying mechanism, namely impressions of fitness. If facial age appearance influences hiring decisions via impressions of fitness, then targeting this mediating process should help to circumvent age discrimination. More specifically, information about candidates’ fitness may counteract the otherwise detrimental impact of an older facial appearance by attenuating or even eliminating the influence of appearance-related fitness impressions. Information that signals candidates’ capability should increase older-looking candidates’ perceived hireability compared to when no information about candidates’ capability is presented. Moreover, no such effect is expected for younger-looking candidates. We expected this to be the case for information about the cognitive fitness of older-looking candidates (capability in an activity that does not obviously require physical fitness) but to be more pronounced for physical fitness (capability in an activity that obviously requires high levels of physical fitness), compared to when no information about older-looking candidates’ capability is presented (Hypothesis 3).

Study 1 uses a sample of lay persons who live in the United States. In Study 2, we recruited Human Resource (HR) professionals from three countries (Austria, Germany, and Switzerland) in order to increase the generalizability of the findings of Study 1. In addition, we tested the context-dependency of the effects of facial age appearance on hireability. Previous research has shown that the fit between various aspects of age and specific job demands affects the degree of age bias (Gordon and Arvey, 2004). For instance, age discrimination based on candidates’ chronological age is particularly prevalent in organizations characterized by rapid change, that is, organizations that have grown and expect to grow rapidly, which highlights the incongruence of stereotypes about older candidates and job requirements (Diekman and Hirnisey, 2007). Thus, it is reasonable to assume that facial age appearance plays a more crucial role in some professional contexts, for instance, when a candidate’s physical appearance is salient, as in a front office job. In this context, we expect that there may be greater perceived incongruence of an older facial age appearance and the job requirements of an appealing physical appearance, leading to increased age discrimination. Whereas managers ascribe to older workers better soft skills in costumer contact because of their experience-based know-how (North and Hershfield, 2014), the fact that first impressions from facial qualities can be formed without awareness (Willis and Todorov, 2006), led us to expect those to take precedence over a more considered evaluation of experience-based know-how.

We therefore manipulated the salience of physical appearance for the job, with high salience of appearance indicated by a job with customer contact, and low salience indicated by a job with no customer contact. We predicted that the tendency for older-looking candidates to receive less favorable hiring ratings than younger-looking ones would be stronger when appearance has high salience than when it has low salience (Hypothesis 4).

Moreover, we considered the gender of job candidates and the age of judges. Past research has not revealed clear evidence for a double standard of appearance for older women vs. men. In a recent field study, age discrimination against older women was found to be more robust compared to age discrimination against older men (Neumark et al., 2015), whereas experimental research failed to show a consistent double standard in hiring (Kaufmann et al., 2016). Research also has revealed that older adults showed greater positivity in their evaluations of older people than did younger adults (Gordon and Arvey, 2004; Zebrowitz and Franklin, 2014). We took both variables into consideration in our analyses.

## Study 1

### Method

#### Participants

The study was conducted online via MTurk. The final sample consisted of 383 participants from the United States (174 women and 209 men; aged between 18 and 77 years; *M*age = 35.44, *SD* = 11.20). Two participants were excluded, because they did not correctly answer the careless responder item (“Please choose “not at all” to answer this question”). The majority, that is, 80% (*n* = 306) of the participants were employed. Thirteen percent of the participants (*n* = 48) were students, 78% (*n* = 298) had a certificate in higher education (secondary school or higher), 6% (*n* = 21) had no school-leaving certificate, and 3% (*n* = 10) gave no information about their educational background.

#### Experimental Design

The experiment was a 2 (Candidates’ Facial Age Appearance: older, younger) × 2 (Candidates’ Chronological Age: older, younger) × 3 (Candidates’ Capability: physical fitness, cognitive fitness, no fitness information) between-subjects design with perceived fitness and hireability as dependent variables. Participants were randomly assigned to the experimental conditions.

#### Procedure

The advertised job was travel agent, an occupation that is perceived as equally suitable for younger and older candidates as well as for men and women (see for example, Kaufmann et al., 2016). The job advertisement contained the job title (travel agent), the name of the travel agency, the educational degree required, and the main tasks of the future incumbent. Participants read a job advertisement and the short résumé of one qualified male or female candidate, which contained information about the candidate’s chronological age and a photo depicting the candidates facial age-appearance, and the candidate’s hobby. We included three men and three women as candidates to increase the generalizability of our findings. The name on the résumé was either male (Mr. Peter Keller) or female (Mrs. Petra Keller). Participants were informed that the short résumés included only essential information. After reviewing the job advertisement and the résumé, participants responded to the manipulation checks, evaluated the candidate, and provided demographic information about themselves. Finally, they were debriefed and thanked for their participation.

##### Age information

**FIGURE 1:**
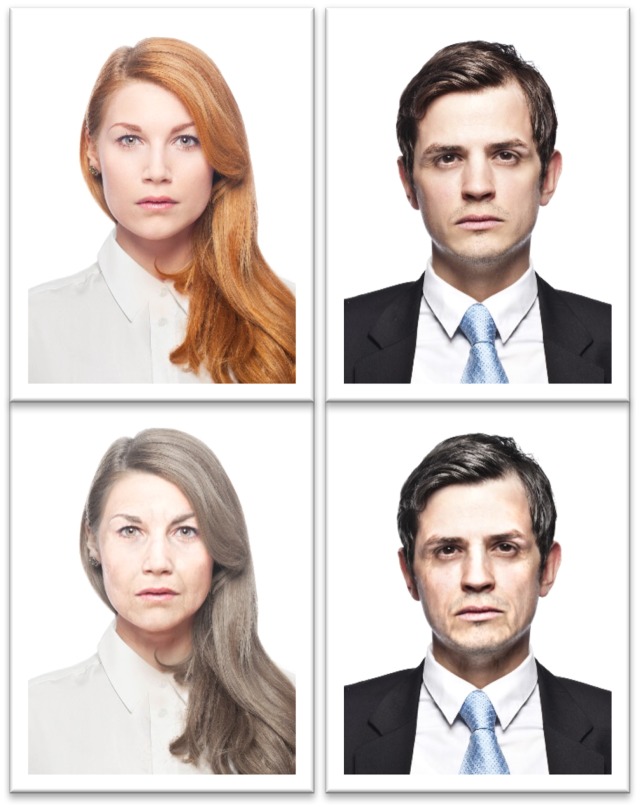
Examples of photographs that were used in the experiment. The younger and the older morph of one male and one female stimulus person are shown.

Chronological age was manipulated by specifying the candidate’s birth date and age (50 or 30 years). Facial age appearance was manipulated by including a photograph of the candidate in the résumé. We used the six photographs (three men, three women) to manipulate facial age appearance. Specifically, we obtained photographs of three women and three men looking 30 years old from iStockphoto and morphed them with April Age to produce morphs that looked roughly 50 years old yielding a total of 12 photographs. Pretesting established that the photographs of all ages did not differ in perceived attractiveness or likeability, while they were perceived as young- or old-looking as intended (30 years vs. 50 years (see **Figure 1**; see pretest in Kaufmann et al., 2016).

##### Capability information

Candidate’s capability was manipulated by indicating fitness of the candidate on the résumé. To include extracuriccular activities (e.g., hobbies) in a résumé is recommended by job search platforms since they signal skills that might be important for future work activities (e.g., www.manpower.ch/en/the-cv). Specifically, we manipulated candidates’ capability by indicating winning the fifth place in their age category in a cooking hobby contest (cognitive fitness information) or in a marathon hobby contest (physical fitness information), or provided no information about fitness (control condition). We chose these hobbies based on a pretest to identify hobbies that might increase perceptions of older-looking candidates fitness. Participants (39 women and 55 men; aged between 18 and 57 years; *M*_age_ = 30.39, *SD*_age_ = 7.91) evaluated in random order the photographs of the six older-looking candidates combined with one of six hobbies (marathon running, cooking, puzzling, golf, craftwork, writing) or with no hobby. Evaluations were made on the 10 items measuring fitness impressions that are described below with the following instruction: “Please rate your first impression of the person concerning the following aspects” (1 = *not at all*, 7 = *very much*). Compared to no hobby, marathon increased physical fitness ascriptions (*t*(22) = 2.89, *p* = 0.005, *d* = 1.25), and cooking slightly increased cognitive fitness ascriptions (*t*(22) = 1.48, *p* = 0.077, *d* = 0.55). Based on these pretest results, we choose cooking to provide information about the candidate’s cognitive fitness and running marathons to provide information about physical fitness. Moreover, to make the hobbies more salient, we described candidates as earning fifth place in his or her age category in a contest, with no information about hobbies and contest provided in the control condition.

##### Fitness impressions

Because there are no validated scales assessing physical and cognitive fitness impressions from faces, we developed our own scales. In another pre-test, we asked participants (18 women and 17 men; aged between 20 and 55 years; *M*_age_ = 25.11, *SD*_age_ = 7.50) to list all terms they could think of to describe physical fitness or cognitive fitness as evident in a face. From this word list, we selected the five most frequently mentioned terms for physical fitness (*N* ≥ 10), namely physically fit, athletic, vital, active, and energetic, and the five most frequently mentioned terms for cognitive fitness (*N* ≥ 10), namely cognitively fit, cognitively active, intelligent, attentive, and interested, and used them in our pretest and both experiments.

In the main experiment of Study 1, responses to these 10 items were measured on seven-point Likert scales, with the following instruction: “Please rate your first impression of the candidate concerning the following aspects” (1 = *not at all*, 7 = *very much*). A principal component factor analysis confirmed that these items could be combined into a single scale (all factor loadings ≥ 0.71; eigenvalue of the first factor 5.86; 59% of explained variance) that captured perceivers’ fitness impressions of the candidate (Cronbach’s α = 0.92).

##### Hireability assessments

To measure *hireability* we asked three questions that capture person-job fit: “To what extent does this applicant fit the demands of the job?,” “To what extent will other employees think this candidate is qualified to do the job?,” “How confident are you that this applicant is qualified for the job?” (Kristof-Brown, 2000). A fourth question assessed hiring intentions: “More than 60 applications were submitted. However, only a small number of applicants can be invited for the job interview. Would you invite this candidate for an interview?” Responses were given on seven-point Likert scales (1 = *not at all/definitely not*, 7 = *very much/definitely yes*), and combined into one scale (Cronbach’s α = 0.91).

### Results

We conducted a preliminary analysis of the data to explore whether candidates’ gender or participants’ age had an impact on the results. That is, we conducted a 2 (Candidates’ Facial Age Appearance: older, younger) × 2 (Candidates’ Chronological Age: older, younger) × 3 (Candidates’ Capability: physical fitness, cognitive fitness, no fitness information) × 2 (Candidates’ Gender: female, male) multivariate analysis of covariance (MANCOVA) with fitness impressions and hireability ratings as dependent variables and age of participants as covariate. We found no significant main effect of candidates’ gender or any interactions of gender with candidates’ age. Moreover, all effects held true when age of participants was used as covariate (see Supplementary Table 1).

We then conducted the main analysis, a 2 (Candidates’ Facial Age Appearance: older, younger) × 2 (Candidates’ Chronological Age: older, younger) × 3 (Candidates’ Capability: physical fitness, cognitive fitness, no fitness information) multivariate analysis (MANOVA) with fitness impressions and hireability ratings as dependent variables. Means and standard deviations are displayed in **Table 1** and statistical effects in **Table 2**.

**Table 1:** Study 1. Means and standard deviations of hireability and fitness impressions by candidates’ facial age appearance, chronological age, and capability information.

				Hireability		Fitness impressions	
Facial age appearance	Chronological age	Capability information	*N*	Mean	*SD*	Mean	*SD*
Younger-looking	Younger	No fitness information	34	6.07	0.84	5.49	0.75
		Cognitive fitness information	28	5.96	0.70	5.44	0.70
		Physical fitness information	31	6.30	0.57	6.03	0.63
	Older	No fitness information	30	6.28	0.72	5.71	0.71
		Cognitive fitness information	33	5.87	1.01	5.30	0.79
		Physical fitness information	29	6.16	0.96	6.01	0.80
Older-looking	Younger	No fitness information	28	5.91	0.70	4.83	0.81
		Cognitive fitness information	39	5.68	0.92	5.12	0.75
		Physical fitness information	32	6.03	0.99	5.59	0.87
	Older	No fitness information	29	5.51	0.95	4.81	0.69
		Cognitive fitness information	35	6.08	0.74	5.16	0.94
		Physical fitness information	35	6.02	0.80	6.08	0.64

**Table 2:** Study 1. Statistical effects of MANOVAs and ANOVAs analyzing hireability and fitness impressions by candidates’ facial age appearance, chronological age, and capability information.

	Multivariate tests						Univariate tests				
	Dependent						Dependent				
	variables	*Wilks’* λ	*F*	df	*p*	η^2^	variables	*F*	df	*p*	η^2^
Candidates’ Facial Age Appearance	Fitness impressions and hireability	0.93	13.04	(2/370)	0.000	0.07	Fitness impressions	25.97	(1/371)	0.000	0.07
							Hireability	7.29	(1/371)	0.007	0.02
Candidates’ Chronological Age	Fitness impressions and hireability	0.99	1.28	(2/370)	0.278	0.01	Fitness impressions	1.59	(1/371)	0.208	0.00
							Hireability	0.00	(1/371)	0.965	0.00
Capability Information	Fitness impressions and hireability	0.82	19.50	(4/740)	0.000	0.10	Fitness impressions	34.93	(2/371)	0.000	0.16
							Hireability	2.60	(2/371)	0.075	0.01
Candidates’ Facial Age Appearance ^∗^ Candidates’ Chronological Age	Fitness impressions and hireability	1.00	0.68	(2/370)	0.509	0.00	Fitness impressions	0.87	(2/371)	0.352	0.00


							Hireability	0.00	(2/371)	0.996	0.00
Candidates’ Facial Age Appearance ^∗^ Capability Information	Fitness impressions and hireability	0.97	3.20	(4/740)	0.013	0.02	Fitness impressions	5.81	(2/371)	0.003	0.03


							Hireability	2.05	(2/371)	0.130	0.01
Candidates’ Chronological Age ^∗^ Capability Information	Fitness impressions and hireability	0.98	2.32	(4/740)	0.056	0.01	Fitness impressions	1.16	(2/371)	0.314	0.01
							Hireability	0.86	(2/371)	0.426	0.01
Candidates’ Facial Age Appearance ^∗^ Candidates’ Chronological Age ^∗^ Capability Information	Fitness impressions and hireability	0.97	2.50	(4/740)	0.041	0.01	Fitness impressions	1.81	(2/371)	0.166	0.01


							Hireability	3.42	(2/371)	0.034	0.02

As expected, we found a significant overall effect of candidates’ facial age appearance [*Wilks’* λ = 0.93, *F*(2,370) = 13.04, *p* = 0.000, η^2^ = 0.07]. Older-looking candidates were perceived as less fit than younger-looking candidates, *F*(1,371) = 25.97, *p* = 0.000, η^2^ = 0.07, and older-looking candidates also received less favorable hireability ratings than younger-looking ones, *F*(1,371) = 7.29, *p* = 0.007, η^2^ = 0.02.

We also found a significant overall effect of information about the candidate’s capability [*Wilks’* λ = 0.82, *F*(4,740) = 19.50, *p* = 0.000, η^2^ = 0.10], with an univariate effect on fitness impressions only, *F*(2,371) = 34.93, *p* = 0.000, η^2^ = 0.16. Planned comparisons revealed that providing physical fitness information (running marathons) increased fitness perceptions of candidates, compared to providing no additional information (control), *t*(246) = -6.91, *p* < 0.001, *d* = 0.89, or cognitive fitness information (cooking), *t*(260) = -7.12, *p* < 0.001, *d* = 0.89. Moreover, fitness perceptions of candidates with cognitive fitness information did not differ from those with no fitness information, *t*(256) = -0.10, *p* = 0.920, *d* = 0.01. Moreover, the effect of candidate’s capability on hireability ratings only approached significance, *F*(2,371) = 2.60, *p* = 0.075, η^2^ = 0.01. Nevertheless, planned comparisons between conditions to test our specific hypothesis revealed that candidates who gave physical fitness information were perceived as more hireable than candidates who gave no fitness information, *t*(246) = 6.91, *p* < 0.001, *d* = 0.89, or who gave cognitive fitness information, *t*(260) = 7.12, *p* < 0.001, *d* = 0.89, while there was no difference in hireability ratings between those with cognitive fitness information and no fitness information, *t*(254) = 0.10, *p* = 0.920, *d* = 0.01.

We also found a significant multivariate interaction of facial age appearance and candidates’ capability [*Wilks’* λ = 0.97, *F*(4,740) = 3.20, *p* = 0.013, η^2^ = 0.02], with a univariate effect for fitness impressions only, *F*(2,371) = 5.81, *p* = 0.003, η^2^ = 0.03, but not for hireability ratings, *F*(2,371) = 2.05, *p* = 0.130, η^2^ = 0.01. Older-looking candidates compared to younger-looking ones, were perceived as less fit when no fitness information was provided, *t*(119) = 5.76, *p* = 0.000, *d* = 1.05. However, facial age appearance had no significant effect on fitness ratings when candidates gave physical fitness information, *t*(125) = 1.32, *p* = 0.191, *d* = -0.24, or cognitive fitness information, *t*(133) = 1.63, *p* = 0.106, *d* = -0.30.

Finally, an unexpected significant three-way interaction between facial age appearance, chronological age, and capability emerged for hireability ratings [*Wilks’* λ = 0.97, *F*(4,740) = 2.50, *p* = 0.041, η^2^ = 0.01]. However follow-up Scheffé tests revealed no significant differences.

All other effects in the MANOVA were non-significant, *F*s ≤ 2.32, *p*s ≥ 0.056, η^2^ ≤ 0.01, showing that also candidates’ chronological age had no effect on fitness impressions nor hireability ratings; *Wilks’* λ = 0.99, *F*(2,370) = 1.28, *p* = 0.278, η^2^ = 0.01.

**FIGURE 2:**
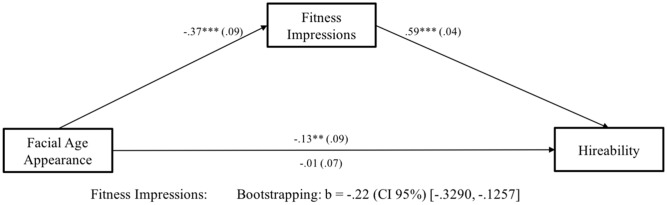
Study 1. *N* = 383. Facial Age Appearance was coded as 0 = younger-looking, 1 = older-looking. Numbers are unstandardized beta-coefficients, with the standard errors shown in parentheses. ^+^*p* < 0.10; ^∗^*p* < 0.05; ^∗∗^*p* < 0.01; ^∗∗∗^*p* < 0.001.

Next, to test whether it was the effect of an older appearance on fitness impressions that led to reduced hireability ratings for older-looking candidates (Hypothesis 2), we conducted a mediation analysis (Hayes, 2013) with 5,000 iterations, and calculated accelerated confidence intervals (CI 95%). Results are depicted in **Figure 2**. As predicted, we found a significant indirect effect of facial age appearance on hireability that was mediated by fitness impressions. Older-looking candidates evoked less favorable fitness impressions, and this reduced hireability ratings for older-looking candidates compared to younger-looking ones, thus confirming Hypothesis 2.

**FIGURE 3:**
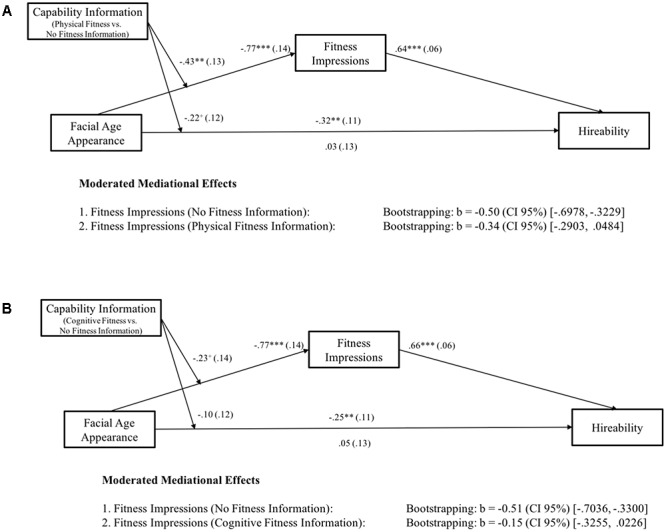
**(A,B)** Study 1. **(A)** *N* = 248 and **(B)** *N* = 256. Facial age appearance was coded as 0 = younger-looking, 1 = older-looking. Capability Information was coded in **(A)** as 0 = no fitness information, 1 = physical fitness information and in **(B)** as 0 = no fitness information, 1 = cognitive fitness information. Numbers are unstandardized beta-coefficients, with the standard errors shown in parentheses. ^+^*p* < 0.10; ^∗^*p* < 0.05; ^∗∗^*p* < 0.01; ^∗∗∗^*p* < 0.001.

Finally, we tested whether information about the candidate’s capability provided within the résumé led to more favorable fitness impressions for older-looking candidates and hence to an increase in perceived hireability (Hypotheses 3). To this end, we calculated two moderated mediation analyses (physical fitness information vs. no fitness information and cognitive fitness information vs. no fitness information as moderator variables) with 5,000 iterations, and calculated accelerated confidence intervals (CI 95%; Hayes, 2013). First, physical fitness information compared to no fitness information was included as moderator for the effect of facial age appearance on fitness impressions and, in turn, on hireability ratings. As predicted (see **Figure 3A**), we found that the indirect effect of facial age appearance on hireability via fitness lost significance when physical fitness information was included compared to when no fitness information was included. Moreover, the indirect effect of facial age appearance on hireability via fitness impressions also lost significance when cognitive fitness information compared to no fitness information was included (see **Figure 3B**).

### Discussion

Results of Study 1 demonstrate that facial age appearance impacts hireability ratings via impressions of fitness: older-looking candidates received less favorable hireability ratings compared to younger-looking ones because they were perceived as less physically and cognitively fit. This mediation effect was altered if candidates’ hobbies provided clear information about their cognitive fitness or their physical fitness. More specifically, if candidates indicated on their résumé that they engaged in award winning cooking or marathon running, an older age appearance no longer decreased perceived fitness or hireability. Moreover, we did not find any effects of chronological age on hireability nor fitness ratings.

Whereas Study 1 showed that capability information moderates effects of facial age-appearance on hireability, Study 2 examined moderation by the job context. More specifically, we investigated to what extent the salience of appearance for a job alters the effects of facial age appearance at hiring. In Study 2 we tested our hypotheses in a sample of HR professionals.

## Study 2

### Method

#### Participants

Participants were 264 HR professionals (121 women and 143 men; aged between 18 and 72 years; *M*_age_ = 42.76, *SD* = 11.07) who were recruited with the help of Qualtrics online panels^1^. The data were collected in Austria, Germany, and Switzerland. Eighty-five percent of the participants worked full time, 76% had a leadership position with a mean of 301 subordinate employees. On average, participants had 11 years of experience in HR and had conducted 36 job interviews over the last 5 years.

#### Experimental Design

The experiment was a 2 (Candidates’ Facial Age Appearance: older, younger) × 2 (Candidates’ Chronological Age: older, younger) × 2 (Salience of Appearance for the Job: front office, back office) between-subjects design with perceived fitness and hireability as dependent variables. Participants were randomly assigned to the experimental conditions.

#### Procedure

The experiment was conducted online, and participants were randomly assigned to the different conditions. Participants read a job advertisement and the short résumé of one qualified male or female candidate, which contained the two age manipulations. As in Study 1, chronological age was manipulated by specifying the candidate’s birth date and age and facial age appearance was manipulated by including a photograph of the candidate in the résumé.

The advertised job was again travel agent. To manipulate the salience of appearance for a job of we used two different versions of the job advertisement, one involving more front-office activities and one involving more back-office work. One version of the job advertisement stated that “The applicant will work in the front office in the flagship store.” (high salience of appearance), while the other version claimed that “The applicant will work in the back office without customer contact.” (low salience of appearance).

We used the same measures for the dependent variables as in Study 1. Again a principal component factor analysis confirmed a single scale for the 10 fitness impressions (all factor loadings ≥ 0.75; eigenvalue of the first factor 6.79; 68% of explained variance; Cronbach’s α = 0.95). The four hireability measures also were combined into one scale (Cronbach’s α = 0.88). In Study 2 we also asked participants “Would you hire the candidate if you had to decide solely on the basis of the documents available?” providing a dichotomous choice (1 = *yes*, 2 = *no*).

### Results

Again a preliminary analysis of the data was conducted to explore whether candidates’ gender or participants’ age had an impact on the results. We conducted a 2 (Candidates’ Facial Age Appearance: older, younger) × 2 (Candidates’ Chronological Age: older, younger) × 2 (Salience of Appearance for the Job: front office, back office) × 2 (Candidates’ Gender: female, male) MANCOVA with fitness impressions and hireability ratings as dependent variables and age of participants as a covariate. Again, we found no significant main effect of candidates’ gender, and no significant interactions of candidates’ gender with candidate’s age. Also all effects held true when age of participants was used as covariate (see Supplementary Table 2).

We then conducted a 2 (Candidates’ Facial Age Appearance: older, younger) × 2 (Candidates’ Chronological Age: older, younger) × 2 (Salience of Appearance for the Job: front office, back office) MANOVA with fitness impressions and hireability as dependent variables. Means and standard deviations are displayed in **Table 3** and statistical effects in **Table 4**.

**Table 3:** Study 2. Means and standard deviations of hireability, fitness impressions and hiring decision by candidates’ facial age appearance, chronological age, and the salience of appearance for the job.

						Fitness		Hiring	
				Hireability		impressions		decision	
Facial age appearance	Chronological age	Salience of appearance for the job	*N*	*M*	*SD*	*M*	*SD*	N (yes)	N (no)
Younger-looking	Younger	Back office	32	5.03	1.35	4.50	1.32	13	19
		Front office	35	5.33	1.11	5.07	1.18	23	12
	Older	Back office	30	4.95	1.02	4.71	0.93	8	22
		Front office	35	5.01	1.37	5.06	1.20	23	12
Older-looking	Younger	Back office	37	4.75	1.09	4.45	1.27	19	18
		Front office	30	4.75	1.36	4.36	1.41	14	15
	Older	Back office	32	5.07	0.94	4.79	0.93	15	17
		Front office	33	4.64	1.23	4.39	1.08	11	22

**Table 4:** Study 2. Statistical effects of MANOVAs and ANOVAs analyzing hireability, fitness and hiring decision by candidates’ facial age appearance, chronological age, and the salience of appearance for the job.

	Multivariate tests						Univariate tests				
	Dependent						Dependent				
	variables	*Wilks’* λ	*F*	df	*p*	η^2^	variables	*F*	df	*p*	η*^2^*
Candidates’ Facial Age Appearance	Fitness impressions and hireability	0.98	2.78	(2/255)	0.064	0.02	Fitness impressions	5.37	(1/256)	0.021	0.02
							Hireability	3.55	(1/256)	0.061	0.01
Candidates’ Chronological Age	Fitness impressions and hireability	0.99	1.31	(2/255)	0.273	0.01	Fitness impressions	0.98	(1/256)	0.324	0.00
							Hireability	0.10	(1/256)	0.758	0.00
Salience of Appearance for the Job	Fitness impressions and hireability	1.00	0.64	(2/255)	0.530	0.01	Fitness impressions	0.59	(1/256)	0.442	0.00
							Hireability	0.01	(1/256)	0.912	0.00
Candidates’ Facial Age Appearance ^∗^ Candidates’ Chronological Age	Fitness impressions and hireability	1.00	0.66	(2/255)	0.516	0.01	Fitness impressions	0.09	(1/256)	0.760	0.00


							Hireability	1.07	(1/256)	0.301	0.00
Candidates’ Facial Age Appearance ^∗^ Salience of Appearance for the Job	Fitness impressions and hireability	0.98	3.03	(2/255)	0.050	0.02	Fitness impressions	5.95	(1/256)	0.015	0.02


							Hireability	1.79	(1/256)	0.182	0.01
Candidates’ Chronological Age ^∗^ Salience of Appearance for the Job	Fitness impressions and hireability	1.00	0.65	(2/255)	0.523	0.01	Fitness impressions	0.83	(1/256)	0.364	0.00


							Hireability	1.25	(1/256)	0.264	0.01
Candidates’ Facial Age Appearance ^∗^ Candidates’ Chronological Age ^∗^ Salience of Appearance for the Job	Fitness impressions and hireability	1.00	0.06	(2/255)	0.944	0.00	Fitness impressions	0.02	(1/256)	0.881	0.00


							Hireability	0.11	(1/256)	0.743	0.00

We found a significant overall effect of facial age appearance as predicted [*Wilks’* λ = 0.97, *F*(2,255) = 2.78, *p* = 0.064, η^2^ = 0.02]. Older-looking candidates compared to younger-looking ones were perceived as less fit, *F*(1,256) = 5.37, *p* = 0.021, η^2^ = 0.02, and tended to receive less favorable hireability ratings, *F*(1,256) = 3.55, *p* = 0.061, η^2^ = 0.01.

We found no overall effects of candidates’ chronological age [*Wilks’* λ = 0.99, *F*(2,255) = 1.31, *p* = 0.273, η^2^ = 0.01], or the salience of appearance for the job [*Wilks’* λ = 1.00, *F*(2,255) = 0.64, *p* = 0.530, η^2^ = 0.01]. However, we found a significant interaction of facial age appearance and salience of appearance for the job [*Wilks’* λ = 0.98, *F*(2,255) = 3.03, *p* = 0.050, η^2^ = 0.02] for fitness impressions, *F*(1,256) = 5.95, *p* = 0.015, η^2^ = 0.023, indicating that older- compared to younger-looking candidates applying for a front office position were perceived as less fit, *t*(131) = 3.30, *p* = 0.000, *d* = 0.57. No such difference in fitness impression emerged for a back office position, *t*(129) = -0.04, *p* = 0.482, *d* = 0.01. All other effects of the MANOVA were not significant, *F* ≤ 1.31, *p* ≥ 0.273, η^2^ ≤ 0.01.

We conducted Chi-Square tests for the dichotomous selection decision. We found that for a front office job, participants were more likely to hire younger-looking candidates (*N* = 46) compared to older-looking ones (*N* = 25; χ^2^ = 6.21, *df* = 1, *p* = 0.013), whereas for a back office job, participants did not differentiate significantly between younger-looking candidates (*N* = 21) and older-looking ones (*N* = 34; χ^2^ = 3.07, *df* = 1, *p* = 0.080). There were no effects of candidates’ chronological age on likelihood of being chosen for a front or back office job (χ^2^ ≥ 1.47, *p* ≤ 0.225).

**FIGURE 4:**
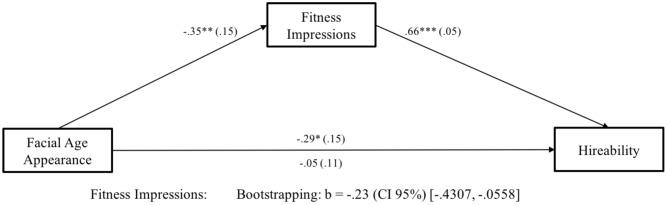
Study 2. *N* = 264. Facial Age Appearance was coded as 0 = younger-looking, 1 = older-looking. Numbers are unstandardized beta-coefficients, with the standard errors shown in parentheses. ^+^*p* < 0.10; ^∗^*p* < 0.05; ^∗∗^*p* < 0.01; ^∗∗∗^*p* < 0.001.

As in Study 1, we conducted a mediation analysis (Hayes, 2013), using bootstrapping technique with 5,000 iterations, and calculating accelerated confidence intervals (CI 95%). Results are depicted in **Figure 4**. As predicted in Hypothesis 2, we found a significant indirect effect of facial age appearance on hireability through fitness impressions. Older-looking candidates evoked less favorable fitness impressions resulting in reduced hireability ratings for older-looking candidates compared to younger-looking ones.

**FIGURE 5:**
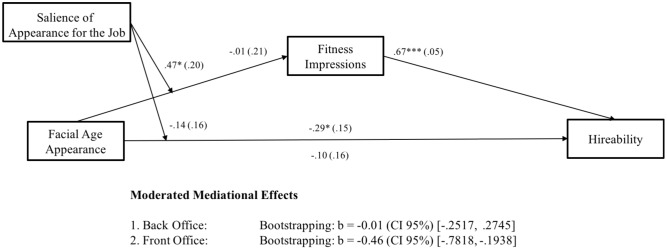
Study 2. *N* = 264. Facial Age Appearance was coded as 0 = young-looking, 1 = old-looking and Salience of Appearance for the Job as 0 = back office, 1 = front office. Numbers are unstandardized beta-coefficients, with the standard errors shown in parentheses. ^+^*p* < 0.10; ^∗^*p* < 0.05; ^∗∗^*p* < 0.01; ^∗∗∗^*p* < 0.001.

Finally, we tested whether greater salience of appearance for the job led to less favorable fitness impressions and hireability ratings for older-looking candidates (see Hypothesis 4). We conducted a moderated mediation analysis with 5,000 iterations, and calculated accelerated confidence intervals (CI 95%; Hayes, 2013). We found an indirect effect for a front office job with customer contact. Specifically, the less favorable hiring ratings for older-looking candidates were mediated by the perception of them as less fit than those who were younger-looking. However, when applying for a back office job, there was neither a direct effect of facial age appearance on hireability nor an indirect effect via fitness impressions (see **Figure 5**).

### Discussion

Replicating results of Study 1, Study 2 revealed that HR professionals perceived older-looking candidates as less fit and less hireable than younger-looking candidates, with the effect of age appearance on hireability mediated by its effect on perceived fitness. Furthermore, this facial age-based discrimination was moderated by the professional context: older-looking candidates evoked less favorable fitness impressions resulting in reduced hireability ratings when applying for a front office job, with a lot of customer contact, where presumably appearance is made salient. In the case of a back office job, there was neither a direct effect of age appearance on hireability nor an indirect effect mediated by fitness impressions. Thus, age appearance effects seem to be activated only when the nature of the job makes appearance salient. The same pattern of results emerged for selection decisions: when participants had to decide if they would hire the candidate, older-looking candidates were chosen less frequently than younger-looking ones but only when applying for the front office job. As in Study 1, we did not find any effects of chronological age on hireability nor fitness ratings.

## General Discussion

This research shows the detrimental effect of an older facial age appearance in selection decisions as well as its underlying mechanism. The results of two experimental studies show that employees as well as HR professionals give lower hireability ratings to older-looking than to younger-looking candidates, because they perceive the former as less fit than the latter. Moreover, this effect persisted, independently of the chronological age of the candidate.

It has been suggested that judgments of more experienced evaluators differ from those of students because the two groups use different criteria (Finkelstein et al., 1995). However, impression formation based on faces is an automatic process (e.g., Hassin and Trope, 2000; Todorov and Uleman, 2003), and facial appearance can evoke trait impressions without the influence of previous experience or knowledge of this process (Freeman and Ambady, 2011). This might explain why not only lay persons but also HR experts perceived older-looking candidates as less fit and thus judged their hireability as lower than younger-looking candidates. Our research also showed that facial age appearance has a greater impact on hiring than chronological age. This replicates earlier research (Kaufmann et al., 2016), and confirms that facial age appearance is very salient and vivid information.

Moreover our research showed that job context has an impact on face-based age discrimination. Older-looking candidates were chosen less often and their hireability ratings were lower than younger-looking ones when applying for a front office job with a great deal of customer contact but not when applying for a back office job with no customer contact. This suggests that the negative effects of an older facial appearance are not activated when the job description makes appearance less salient.

Results of this research point to fitness impressions as one central mechanism that underlies the effect of facial age appearance on hiring. This was demonstrated by mediation analyses as well as by direct manipulations of the mediator, i.e., candidates’ fitness (Study 1). More specifically, the results of Study 1 showed that clear information about candidates’ fitness in the résumé buffered the negative effect of an older appearance on fitness impressions and hireability. Interestingly, this buffering was achieved not only by information that specifically targeted a candidate’s physical and cognitive fitness (in this research, running marathons as a hobby) but also by more general information about cognitive fitness (in this research, award winning cooking as a hobby). Both circumvented the face-based age bias by reducing negative fitness impressions which in turn increased hireability ratings of older-looking candidates. The ameliorative effect of the cooking hobby might be related to early research showing that effects of diagnostic information (like age appearance) are mitigated by irrelevant individuating information, which has been called the dilution effect (Nisbett et al., 1981).

Taken together, the results of this research point to the importance of differentiating between candidates’ chronological age and age-appearance when investigating age discrimination in personnel selection procedures. Whereas candidates’ facial age appearance was found to drive age discrimination explained by negative fitness impressions of an older appearance, chronological age did not explain older candidates disadvantages in personnel selection. Therefore, new directions for models of age discrimination are demanded. Whereas models of age discrimination claim that chronological age triggers age stereotyping which in turn results in discrimination, current models of person construal (e.g., Freeman and Ambady, 2011) propose that discrimination is a product of the joint influence of category information (e.g., chronological age) and sensory cues (e.g., facial age appearance). We therefore investigated, for the first time, both sources of age information in combination and found that the influence of facial age appearance exceeds that of chronological age. Moreover, we identified one key mechanism of facial-age based discrimination by showing that an older facial age appearance triggers lower fitness impressions resulting in less favorable hireability ratings for older-looking candidates compared to younger-looking ones. Furthermore, we also documented that the impact of an older facial age appearance on hireability depended on the professional context, namely specific job requirements (back versus front office). Taken together our results indicate that current models of age discrimination need to be extended by taking the effects of facial age information and its underlying mechanisms into account.

### Reducing Face-Based Age Discrimination at Hiring

The results of Study 1 suggest that job candidates themselves can use a strategy to reduce age discrimination: providing counter-stereotypic information to refute potentially prejudiced impressions in recruiters (e.g., Heilman and Okimoto, 2007). It has been observed that older job applicants who are aware of age-related biases attempt to signal skills their age group is believed to lack or to change their appearance to look more youthful (Berger, 2009). However, such strategies combat only the symptoms and not the cause of age discrimination in personnel selection. Therefore, not only do candidates themselves need strategies to prevent discrimination, but also organizations need measures to create a discrimination-free environment in recruitment (Spencer et al., 2016).

One promising organizational strategy to reduce face-based age discrimination at hiring would be to exclude photographs from résumés, so that there is no information on facial-age appearance, at least during the first phase of résumé screening. Indeed, chronologically older and older-looking candidates were found to have similar chances of employment as did both chronologically younger and younger-looking candidates in anonymous application procedures (Kaufmann et al., 2016). In some countries, there is already a tendency to use résumés without photographs in recruitment suggesting that the classic application photograph will become less important in the future (Weitzel et al., 2015). But at the same time, the problem of facial age-based discrimination survives in modern forms of recruitment, where candidates’ photographs are gaining importance, for instance, in social media like LinkedIn, where candidates are encouraged to upload a photograph in order to be successful.

### Limitations and Directions for Future Research

In past research, when candidate’s chronological age and facial age appearance were examined independently of each other, only an older facial appearance, but not a chronological older age had a negative effect on being selected for an interview (Kaufmann et al., 2016). In the present research, detrimental effects of an older facial age appearance on hireability persisted, independently of the chronological age of the candidate. One explanation might be that chronological age is less salient and vivid, although, we tried to make candidate’s chronological age as salient as possible by including date of birth as well as years of life in parentheses. Additionally, age biases are generally weak (e.g., Kite et al., 2005) and we described all candidates in the fictitious résumés as highly qualified (e.g., detailed information about qualifications and work experiences). Another possibility is that people made an effort not to discriminate based on age. Whereas this may engage controlled processing that eliminates discrimination based on chronological age, it is less likely to mitigate discrimination based on age-appearance which involves automatic processing. Finally, earlier research has shown that most jobs are associated with a specific (implicit) age norm (Lawrence, 1988). Age norms that favor younger workers are found to be strongly associated with industries such as finance, insurance, retailing, and information technology/computing (Arrowsmith and McGoldrick, 1996; Perry and Finkelstein, 1999). If older workers apply for a job with a younger age norm, they will be more likely to face age discrimination (Perry et al., 1996). Thus, it might be that not only older-looking candidates but also chronologically older candidates will receive less favorable hireability ratings compared to younger candidate if the respective job is associated with a younger age norm. Nevertheless, more research is needed about the influence of chronological age and facial age appearance in hiring decisions for different jobs in different industries.

We did not find a double standard of aging for older women compared to older men in hireability ratings, which some previous research suggests might be expected due to the so-called double standard of aging: women are more readily categorized as “old” than men are. As a consequence (negative) age stereotypes hit women at a younger age than men (e.g., Kite et al., 2005). Older women are also perceived as less attractive than men of the same age (McKelvie, 1993; McLellan and McKelvie, 1993; but for an exception see Zebrowitz et al., 1993 who compared the same people across the lifespan). Our study may have been insensitive to the double standard of aging because we choose photographs of older and younger candidates who were perceived as equal in both attractiveness and likeability (Kaufmann et al., 2016). However, this methodology has the strength of ruling out the possibility that older-looking applicants were judged as less hireable simply because they were less attractive or less likeable.

We only used photographs of Caucasians in our studies. Like age-related facial qualities, those related to ethnicity lead to categorization and are directly associated with stereotypic traits that may affect perceived fitness for a job (e.g., Maddox and Gray, 2002). It remains to be determined whether the effects of facial age appearance and the null effects of chronological age would generalize to different ethnic groups.

Finally, future research should investigate ageist attitudes as potential moderators of face-based age discrimination, as meta-analytical research showing that ageist attitudes and age stereotypes can produce stronger discriminatory reactions to older workers in organizational settings (Kite and Johnson, 1988; Finkelstein et al., 1995; Gordon and Arvey, 2004).

## Conclusion

The present research underlines the importance of facial age appearance at hiring. Results of two experimental studies demonstrate the detrimental effects of an older facial appearance that exist independently of candidates’ chronological age, and that are driven by unfavorable fitness impressions of older-looking candidates. However, although older faces automatically evoke unfavorable fitness impressions, which have a negative impact on hiring decisions, our research also shows that this negative impact is most pronounced for jobs where appearance is salient and that it is possible to reduce face-based age discrimination by providing positive information about candidates’ fitness and capability. One important implication of our findings is that removing photographs from applications may eliminate age discrimination in the first phase of a recruitment process or before employers see a candidate’s social media profile. Our results further imply that older candidates can take steps to increase their hireability by including personalizing information on their résumé, particularly those demonstrating physical or cognitive fitness.

## Ethics Statement

Prior to data collection, the ethical committee of the University of Lausanne (Faculty of Business and Economics) approved both studies as being risk-free for participants and as maintaining their anonymity.

## Author Contributions

MK, FK, and SS developed the initial research idea and the concrete study concept was generated by those authors. MK performed the data analysis. MK together with FK and LZ interpreted the results. MK drafted the manuscript, and FK, SS, and LZ provided critical revisions. All authors approved the final version of the manuscript for submission.

## Conflict of Interest Statement

The authors declare that the research was conducted in the absence of any commercial or financial relationships that could be construed as a potential conflict of interest.

## Footnotes

1 http://www.qualtrics.com/panel-management/
