# Supplementary material for: Age Bias in Selection Decisions: The Role of Facial Appearance and Fitness Impressions
Source: Front Psychol. 2017 Dec 8;8:2065. doi: 10.3389/fpsyg.2017.02065 (PMC5727086; doi:10.3389/fpsyg.2017.02065)
Supplement: Supplementary file 1 [file Table_1.docx]

*Table 1.* Study 1. Statistical effects of MANCOVAs and ANCOVAs analyzing hireability and fitness impressions by candidates’ facial age appearance, chronological age, capability information, and candidates’ gender with age of participants as covariate.

|  | **Multivariate Tests** | | | | | | **Univariate Tests** | | | | | |
| --- | --- | --- | --- | --- | --- | --- | --- | --- | --- | --- | --- | --- |
|  | **Dependent Variables** | ***Wilks’ λ*** | **F** | **df** | **p** | ***η^2^*** | **Dependent Variables** | **F** | **df** | **p** | ***η^2^*** |  |
| Participants’ Age | Fitness Impressions and Hireability | 0.97 | 5.36 | (2/357) | .005 | 0.029 | Fitness Impressions | 10.61 | (1/358) | .001 | 0.03 |  |
|  |  |  |  |  |  |  | Hireability | 4.99 | (1/358) | .026 | 0.01 |  |
| Candidates’ Facial Age Appearance | Fitness Impressions and Hireability | 0.93 | 13.84 | (2/357) | .000 | 0.072 | Fitness Impressions | 27.64 | (1/358) | .000 | 0.07 |  |
|  |  |  |  |  |  |  | Hireability | 8.06 | (1/358) | .005 | 0.02 |  |
| Candidates’ Chronological Age | Fitness Impressions and Hireability | 0.99 | 1.18 | (2/357) | .308 | 0.007 | Fitness Impressions | 1.45 | (1/358) | .230 | 0.00 |  |
|  |  |  |  |  |  |  | Hireability | 0.00 | (1/358) | .950 | 0.00 |  |
| Capability Information | Fitness Impressions and Hireability | 0.81 | 19.87 | (4/714) | .000 | 0.100 | Fitness Impressions | 35.64 | (2/358) | .000 | 0.17 |  |
|  |  |  |  |  |  |  | Hireability | 2.62 | (2/358) | .074 | 0.01 |  |
| Candidates’ Gender | Fitness Impressions and Hireability | 1.00 | 0.88 | (2/357) | .417 | 0.005 | Fitness Impressions | 1.72 | (1/358) | .190 | 0.01 |  |
|  |  |  |  |  |  |  | Hireability | 0.39 | (1/358) | .532 | 0.00 |  |
| Candidates’ Facial Age Appearance * Candidates’ Chronological Age | Fitness Impressions and Hireability | 1.00 | 0.71 | (2/357) | .494 | 0.004 | Fitness Impressions | 0.74 | (1/358) | .389 | 0.00 |  |
|  |  |  |  |  |  |  | Hireability | 0.02 | (1/358) | .880 | 0.00 |  |
| Candidates’ Facial Age Appearance * Capability Information | Fitness Impressions and Hireability | 0.97 | 2.94 | (4/714) | .020 | 0.016 | Fitness Impressions | 5.37 | (2/358) | .005 | 0.03 |  |
|  |  |  |  |  |  |  | Hireability | 1.89 | (2/358) | .153 | 0.01 |  |
| Candidates’ Facial Age Appearance * Candidates’ Gender | Fitness Impressions and Hireability | 1.00 | 0.42 | (2/357) | .655 | 0.002 | Fitness Impressions | 0.85 | (1/358) | .358 | 0.00 |  |
|  |  |  |  |  |  |  | Hireability | 0.26 | (1/358) | .611 | 0.00 |  |
| Candidates’ Chronological Age * Capability Information | Fitness Impressions and Hireability | 0.98 | 2.06 | (4/714) | .085 | 0.011 | Fitness Impressions | 0.92 | (2/358) | .401 | 0.01 |  |
|  |  |  |  |  |  |  | Hireability | 0.83 | (2/358) | .436 | 0.01 |  |
| Candidates’ Chronological Age * Candidates’ Gender | Fitness Impressions and Hireability | 1.00 | 0.34 | (2/357) | .709 | 0.002 | Fitness Impressions | 0.00 | (1/358) | .968 | 0.00 |  |
|  |  |  |  |  |  |  | Hireability | 0.42 | (1/358) | .519 | 0.00 |  |
| Capability Information * Candidates’ Gender | Fitness Impressions and Hireability | 0.99 | 1.39 | (4/714) | .237 | 0.008 | Fitness Impressions | 2.54 | (2/358) | .080 | 0.01 |  |
|  |  |  |  |  |  |  | Hireability | 0.50 | (2/358) | .607 | 0.00 |  |
| Candidates’ Facial Age Appearance * Candidates’ Chronological Age * Capability Information | Fitness Impressions and Hireability | 0.97 | 2.73 | (4/714) | .028 | 0.015 | Fitness Impressions | 2.29 | (2/358) | .103 | 0.01 |  |
|  |  |  |  |  |  |  | Hireability | 3.39 | (2/358) | .035 | 0.02 |  |
| Candidates’ Facial Age Appearance * Candidates’ Chronological Age * Candidates’ Gender | Fitness Impressions and Hireability | 1.00 | 0.06 | (2/357) | .939 | 0.000 | Fitness Impressions | 0.00 | (1/358) | .991 | 0.00 |  |
|  |  |  |  |  |  |  | Hireability | 0.09 | (1/358) | .770 | 0.00 |  |
| Candidates’ Facial Age Appearance * Capability Information * Candidates’ Gender | Fitness Impressions and Hireability | 0.99 | 0.71 | (4/714) | .588 | 0.004 | Fitness Impressions | 0.93 | (2/358) | .396 | 0.01 |  |
|  |  |  |  |  |  |  | Hireability | 0.02 | (2/358) | .983 | 0.00 |  |
| Candidates’ Chronological Age * Capability Information * Candidates’ Gender | Fitness Impressions and Hireability | 0.99 | 1.28 | (4/714) | .278 | 0.007 | Fitness Impressions | 0.14 | (2/358) | .873 | 0.00 |  |
|  |  |  |  |  |  |  | Hireability | 1.53 | (2/358) | .219 | 0.01 |  |
| Candidates’ Facial Age Appearance * Candidates’ Chronological Age *  Capability Information * Candidates’ Gender | Fitness Impressions and Hireability | 1.00 | 0.18 | (4/714) | .947 | 0.001 | Fitness Impressions | 0.27 | (2/358) | .761 | 0.00 |  |
|  |  |  |  |  |  |  | Hireability | 0.26 | (2/358) | .770 | 0.00 |  |

*Table 2.* Study 2. Statistical effects of MANCOVAs and ANCOVAs analyzing hireability and fitness impressions by candidates’ facial age appearance, chronological age, the salience of appearance of the job, and candidates’ gender with age of participants as covariate.

|  | **Multivariate Tests** | | | | | | **Univariate Tests** | | | | |
| --- | --- | --- | --- | --- | --- | --- | --- | --- | --- | --- | --- |
|  | **Dependent Variables** | ***Wilks’ λ*** | **F** | **df** | **p** | ***η^2^*** | **Dependent Variables** | **F** | **df** | **p** | ***η^2^*** |
| Participants’Age | Fitness Impressions and Hireability | 0.97 | 3.40 | (2/246) | .035 | 0.03 | Fitness Impressions | 5.18 | (1/247) | .024 | 0.02 |
|  |  |  |  |  |  |  | Hireability | 0.32 | (1/247) | .574 | 0.00 |
| Candidates’ Facial Age Appearance | Fitness Impressions and Hireability | 0.98 | 2.41 | (2/246) | .092 | 0.02 | Fitness Impressions | 4.70 | (1/247) | .031 | 0.02 |
|  |  |  |  |  |  |  | Hireability | 2.99 | (1/247) | .085 | 0.01 |
| Candidates’ Chronological Age | Fitness Impressions and Hireability | 0.99 | 1.30 | (2/246) | .275 | 0.01 | Fitness Impressions | 1.03 | (1/247) | .311 | 0.00 |
|  |  |  |  |  |  |  | Hireability | 0.07 | (1/247) | .798 | 0.00 |
| Salience of Appearance for the Job | Fitness Impressions and Hireability | 0.99 | 0.76 | (2/246) | .468 | 0.01 | Fitness Impressions | 0.78 | (1/247) | .379 | 0.00 |
|  |  |  |  |  |  |  | Hireability | 0.00 | (1/247) | .955 | 0.00 |
| Candidates’ Gender | Fitness Impressions and Hireability | 1.00 | 0.43 | (2/246) | .650 | 0.00 | Fitness Impressions | 0.86 | (1/247) | .354 | 0.00 |
|  |  |  |  |  |  |  | Hireability | 0.44 | (1/247) | .509 | 0.00 |
| Candidates’ Facial Age Appearance * Candidates’ Chronological Age | Fitness Impressions and Hireability | 1.00 | 0.60 | (2/246) | .550 | 0.01 | Fitness Impressions | 0.01 | (1/247) | .907 | 0.00 |
|  |  |  |  |  |  |  | Hireability | 0.79 | (1/247) | .374 | 0.00 |
| Candidates’ Facial Age Appearance * Salience of Appearance for the Job | Fitness Impressions and Hireability | 0.98 | 3.03 | (2/246) | .050 | 0.02 | Fitness Impressions | 6.00 | (1/247) | .015 | 0.02 |
|  |  |  |  |  |  |  | Hireability | 2.01 | (1/247) | .158 | 0.01 |
| Candidates’ Facial Age Appearance * Candidates’ Gender | Fitness Impressions and Hireability | 1.00 | 0.16 | (2/246) | .850 | 0.00 | Fitness Impressions | 0.17 | (1/247) | .683 | 0.00 |
|  |  |  |  |  |  |  | Hireability | 0.33 | (1/247) | .569 | 0.00 |
| Candidates’ Chronological Age * Salience of Appearance for the Job | Fitness Impressions and Hireability | 0.99 | 0.83 | (2/246) | .438 | 0.01 | Fitness Impressions | 1.10 | (1/247) | .296 | 0.00 |
|  |  |  |  |  |  |  | Hireability | 1.59 | (1/247) | .209 | 0.01 |
| Candidates’ Chronological Age * Candidates’ Gender | Fitness Impressions and Hireability | 0.99 | 1.03 | (2/246) | .360 | 0.01 | Fitness Impressions | 1.85 | (1/247) | .176 | 0.01 |
|  |  |  |  |  |  |  | Hireability | 0.31 | (1/247) | .576 | 0.00 |
| Salience of Appearance for the Job * Candidates’ Gender | Fitness Impressions and Hireability | 0.99 | 1.47 | (2/246) | .231 | 0.01 | Fitness Impressions | 0.05 | (1/247) | .821 | 0.00 |
|  |  |  |  |  |  |  | Hireability | 1.25 | (1/247) | .265 | 0.01 |
| Candidates’ Facial Age Appearance * Candidates’ Chronological Age * Salience of Appearance for the Job | Fitness Impressions and Hireability | 1.00 | 0.05 | (2/246) | .947 | 0.00 | Fitness Impressions | 0.01 | (1/247) | .915 | 0.00 |
|  |  |  |  |  |  |  | Hireability | 0.09 | (1/247) | .761 | 0.00 |
| Candidates’ Facial Age Appearance * Candidates’ Chronological Age * Candidates’ Gender | Fitness Impressions and Hireability | 1.00 | 0.33 | (2/246) | .719 | 0.00 | Fitness Impressions | 0.56 | (1/247) | .455 | 0.00 |
|  |  |  |  |  |  |  | Hireability | 0.54 | (1/247) | .461 | 0.00 |
| Candidates’ Facial Age Appearance * Salience of Appearance for the Job * Candidates’ Gender | Fitness Impressions and Hireability | 1.00 | 0.40 | (2/246) | .668 | 0.00 | Fitness Impressions | 0.06 | (1/247) | .809 | 0.00 |
|  |  |  |  |  |  |  | Hireability | 0.65 | (1/247) | .420 | 0.00 |
| Candidates’ Chronological Age * Salience of Appearance for the Job * Candidates’ Gender | Fitness Impressions and Hireability | 1.00 | 0.41 | (2/246) | .661 | 0.00 | Fitness Impressions | 0.66 | (1/247) | .419 | 0.00 |
|  |  |  |  |  |  |  | Hireability | 0.05 | (1/247) | .820 | 0.00 |
| Candidates’ Facial Age Appearance * Candidates’ Chronological Age * Salience of Appearance for the Job * Candidates’ Gender | Fitness Impressions and Hireability | 1.00 | 0.29 | (2/246) | .747 | 0.00 | Fitness Impressions | 0.06 | (1/247) | .813 | 0.00 |
|  |  |  |  |  |  |  | Hireability | 0.49 | (1/247) | .485 | 0.00 |
